# Evaluation of Transdermal Transport and Concurrent Cutaneous Hydrolysis of Timolol Prodrug for the Treatment of Infantile Hemangiomas

**DOI:** 10.3390/pharmaceutics14081607

**Published:** 2022-08-01

**Authors:** Shih-Jen Chang, Huiyuan Wang, Jialin Chen, Qianyi Chen, Lei Chang, Yongzhuo Huang, Yajing Qiu, Xiaoxi Lin

**Affiliations:** 1 Plastic and Reconstructive Surgery, Shanghai Ninth People’s Hospital, Shanghai Jiao Tong University School of Medicine, Shanghai 200011, China; changshihjen@sjtu.edu.cn (S.-J.C.); chenjl403@sjtu.edu.cn (J.C.); chenqyi@sjtu.edu.cn (Q.C.); changleiray@126.com (L.C.); 2 State Key Laboratory of Drug Research, Shanghai Institute of Materia Medica, Chinese Academy of Sciences, Shanghai 201203, China; wanghuiyuan@simm.ac.cn (H.W.); yzhuang@simm.ac.cn (Y.H.)

**Keywords:** infantile hemangioma, timolol maleate, butyryl timolol maleate, prodrug, sequelae, hydrolysis

## Abstract

Infantile hemangiomas (IH) leave sequelae after involution. Topical application of timolol maleate (TM) is the mainstream treatment for superficial lesions but is limited by its low penetrable properties. We aimed to develop a superior skin permeation drug while maintaining the therapeutic properties of timolol. We predict that this drug will promote the involution of thick and deep IH lesions and avoid sequelae. We chemically modified drug structure to prepare butyryl timolol maleate (BT) prodrug and conducted in vitro and in vivo toxicity evaluations of BT with rat dorsal skin and normal skin cells. Skin permeation and absorption comparisons of TM and BT were conducted using rat and porcine skin models. Conversion efficiency of BT to timolol was also tested on human skin ex vivo. BT did not cause skin irritation on rat dorsal skin and exhibited low cytotoxicity overall. BT exhibited superior skin permeation ability compared with that of TM, whilst maintaining a low systemic absorbance. Further, BT was converted to timolol in human skin in a time-dependent manner. Noticeably, timolol accumulation in the skin from BT was higher than that from TM. Finally, BT demonstrated similar biocompatibility with TM in the IH tumor. BT enhances local delivery of timolol and its skin permeation. Using BT, we could eliminate thicker IH lesions that are prone to leave sequelae, and potentially help young children avoid dermal sequelae, disfigurement, and concomitant therapy.

## 1. Introduction

Infantile hemangiomas (IH) is the most common type of benign tumor on the head and neck of approximately 5% of infants [1,2,3]. The natural course of IH consists of three phases. The first phase is rapid proliferation that occurs shortly after birth, where the tumor grows aggressively and potentially causes dysfunction or disfigurement. This is followed by a stabilization phase for a few months, and finally the involution phase that typically starts at one year old and continues throughout childhood. Although IHs spontaneously involute, different levels of disfigurement can occur in more than half of untreated patients [4,5], leading to potential socially adverse impacts on these children [6].

In 2008, Leaute-Labreze et al. discovered systemic propranolol to be effective in treating IH lesions, and it has since become the first-line therapy for large IHs [7,8]. Most IH lesions are localized and relatively small in size. However, a few studies have reported that propranolol is less effective when administered via intralesional or topical routes [9,10]. For a single superficial IH, topical application of timolol maleate (TM), a non-selective beta-blocker, has been clinically proven a more effective treatment method. Regional use of topical 0.5% TM solution offers more convenience while avoiding the possible systemic effects such as bradycardia and bronchospasm caused by propranolol [11,12].

Though topical usage of TM is prevalent, its limited penetration ability renders it less effective in treating thicker IH lesions. Previous studies have reported that the best response to topical TM application occurs in lesions less than 1-mm thick [12]. However, lesions with over 2-mm thickness are at a greater risk of leaving permanent scarring and skin changes [13]. It has been speculated that skin concentrations of timolol are likely higher in thin hemangiomas, exerting a local therapeutic effect that is lost in thicker, more vascularized hemangiomas [14]. While TM is applied on the surface of hemangiomas as a transdermal drug delivery system, the primary factor limiting the efficiency of drug delivery is its physicochemical properties. Therefore, we decided to chemically modify the drug structure to prepare a prodrug, which may be conducive to better dermal penetration and function.

Noticeably, lipophilic properties enable drug dissolution in the stratum corneum, hydrophilicity facilitates drug distribution from the stratum corneum to the skin. The design of butyryl timolol (BT) combines both these drug properties, for example the lipophilic prodrug BT more easily penetrates the stratum corneum than the original drug TM, after entering the epidermis, it is then immediately converted to the prototype drug timolol, which then acts in the dermis.

Hence, we hypothesize that BT would exhibit a superior skin penetration and timolol biotransformation without affecting the normal skin physiology. These effects could facilitate the use of TM treating deeper IH lesions.

## 2. Materials and Methods

### 2.1. Materials

(S)-Timolol maleate was purchased from CSNpharm (Arlington Heights, IL, USA) and prepared according to the manufacturer’s instructions. Pooled human umbilical vein endothelial cells (HUVEC) were purchased from Promocell (Heidelberg, Germany). Human foreskin fibroblast cells (HFF-1) were kindly provided by the cell bank of the Shanghai Institutes for Biological Sciences. Human skin was obtained with consent (Project of Biobank No. YBKA201902 from Shanghai Ninth People’s Hospital, Shanghai Jiao Tong University School of Medicine) from rhytidectomy patients of the Department of Plastic and Reconstructive Surgery, Shanghai Ninth People’s Hospital. Low glucose and high glucose Dulbecco’s modified Eagle’s medium (DMEM) and phosphate buffer saline (PBS) were purchased from Gibco (New York, NY, USA), endothelial growth medium (EGM-2) from Promocell, and mesenchymal stem cell medium (MSC) was obtained from Basal Media (Shanghai, China). Further, fetal bovine serum (FBS) was purchased from Cellmax (Beijing, China), and a Cell Counting Kit-8 (CCK8) assay was purchased from Dojindo (Rockville, MD, USA).

### 2.2. Pro-Drug BT Synthesis

**Figure 1:**
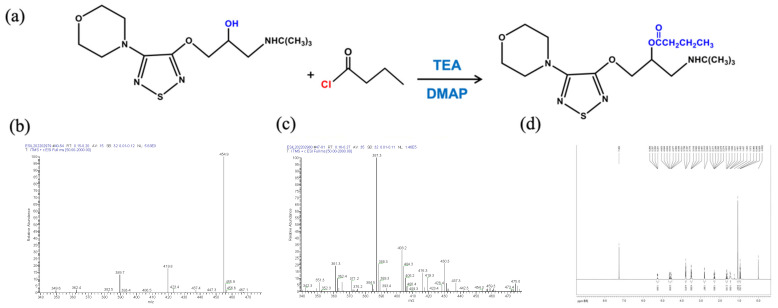
Synthesis and characterization of butyryl timolol (BT). (**a**) Diagram of BT synthesis; (**b**) ESI/MS of Timolol Maleate ™; (**c**) ESI/MS and (**d**) D500-MHz 1H NMR spectra of synthesized BT.

The Pro-drug BT was synthesized from (S)-timolol maleate and butyryl chloride. (S)-Timolol maleate (2.0 g, 4.6 mmol) was dissolved in 10% NaOH (30 mL) and stirred for 30 min, followed by the addition of three 30 mL portions of dichloromethane. The organic layer was dried with anhydrous sodium sulfate and then evaporated to obtain timolol (1.4 g, 96% productivity, oil). Under the protection of argon, timolol (240 mg, 0.76 mmol) was dissolved in dichloromethane (10 mL), triethylamine (115 mg, 1.14 mmol), DMAP (145 mg, 1.14 mmol), and butyryl chloride (121.5 mg, 1.14 mmol). After stirring for 24 h at room temperature, 30 mL of dichloromethane was added, the resulting solution was washed with water and saturated salt, and dried with anhydrous sodium sulfate. The solvent was removed under high vacuum and the residue was collected. This technique was modified from a published protocol [15] with a newly developed reactant molar ratio. The Pro-drug BT was purified by preparative high-performance liquid chromatography. The molecular weights were determined by an electrospray ionization mass spectrometry (LTQ XL™, ThermoFisher Scientific, Waltham, MA, USA), and proton magnetic resonance (1H NMR) was recorded in CDC13 using an AVANCEIII 500 MHz spectrometer (Bruker, Billerica, MA, USA). Figure 1a depicts the diagram of BT synthesis. 

### 2.3. Animals

Three-week old specific pathogen-free (SPF) SD rats were supplied by Shanghai JieSiJie Laboratory Animals Co. Ltd. (Shanghai, China). A one-month-old pig was supplied by the Shanghai Jiagan Biotechnology Co., Ltd. (Shanghai, China). The animal experiment protocol was reviewed and approved by the Animal care & Welfare committee of Shanghai Jiagan Biotechnology (Approval Number LL20210201-1) and the Institutional Animal Care and Use Committee of Shanghai Ninth People’s hospital (Approval Number SH9H-2021-A229-1). Animals were housed and handled according to National Research Council’s Guide for the Care and Use of Laboratory Animals.

### 2.4. Harvesting Infantile Hemangioma Cells

Infantile hemangioma endothelial cells (IHEC), infantile hemangioma stem cells (IHSC), and infantile hemangioma pericytes (IHPC) were isolated from fresh surgical specimens according to the following protocol [16]. Briefly, superficial or mixed infantile hemangioma lesion samples in the proliferative phase were surgically excised and washed with PBS twice, then transported to the lab within an hour in a sterile container with low glucose DMEM. The skin was removed from the lesion with a scalpel and the tumor was diced. IH fragments were collected and transferred into 15-mL centrifuge tubes (Jet Biofil, Guangdong, China) with 0.2% collagenase type 1 (Worthington, Lakewood, NJ, USA) in DMEM and incubated at 37 °C on a 60 r/min rotator for 1 h. The suspension was then filtered through a 70-µm cell strainer (Corning, New York, NY, USA) and centrifuged at 1200 rpm for 5 min. Subsequently, the supernatant was discarded, and CD31, CD133 and CD146 positive cells were isolated from the suspension with MACS magnetic microbeads (Miltenyi Biotec, Bergisch Gladbach, Germany) to obtain IHEC, IHSC, and IHPC, respectively.

### 2.5. Cell Culture Conditions

IHEC, IHSC, and HUVECs were routinely maintained in EGM-2 supplemented with 10% fetal bovine serum and 1% penicillin/streptomycin. IHPC was cultured in MSC with 10% fetal bovine serum and 1% penicillin/streptomycin. HFF-1 was cultured in high-glucose DMEM with 10% fetal bovine serum and 1% penicillin/streptomycin. Cells were incubated in a 5% CO_2_ atmosphere at 37 °C. Media was changed after reseeding 2–3 times a week. Cell detachment was performed by employing trypsin-EDTA solution. Cell culture confluency was approximately 80% confluency at the time of treatment. Results obtained after treating cells with vehicle were strictly comparable to those obtained with untreated cells. IH cells of passages 2–4 were used for this study.

### 2.6. In Vitro and In Vivo Toxicity Evaluations of BT 

#### 2.6.1. Histological Examination of Rat Dorsal Skin after Topical Application

Eighteen (three for each group) three-week-old rats were anesthetized with 8% emulsified isoflurane (0.51–0.64 mL/kg) and randomly divided into six equal groups that received topical 0.9% sodium chloride, 0.5% TM or 0.5% BT treatment for 1 or 4 h. Briefly, each mouse was anesthetized with an intraperitoneal anesthetic injection and placed on its abdomen. A 1 cm by 1 cm square was drawn on its back and a 1 cm by 1 cm gauze soaked with the corresponding application was applied. Preservative film was used to cover the gauze to prevent evaporation. After the appointed time of treatment, the gauze was removed to observe the skin reaction. The squared dorsal skin area was subsequently excised. Specimens were fixed in 4% neutral buffered formalin, paraffin embedded followed by slicing, slide preparation, and staining with hematoxylin and eosin (H&E, Servicebio, Wuhan, China), and examined under a light microscope (Nikon, Tokyo, Japan).

#### 2.6.2. Cytotoxicity of BT in IH and Normal Skin Cells 

HUVEC, HFF-1, IHEC, IHSC, and IHPC were plated onto 96 flat-bottom wells at a density of 4000 cells per well and starved overnight. Cells were treated with multiple concentrations of TM and BT (0–1000 µM) and vehicle in DMEM for 72 h. The CCK8 assay was used to detect cell viability in accordance with the manufacturer’s instructions. Supernatants were discarded and cells were incubated with 10 µL of CCK8. The absorbance at 450 nm was measured using a 96-well plate reader (Synergy H1, Biotek, Winooski, VT, USA). 

### 2.7. Skin Permeation and Skin Absorption Studies

#### 2.7.1. Permeation Studies Using Franz Diffusion Cells

Full-thickness skin samples were excised from the dorsal region of a one-month-old pig. Subcutaneous fat, tissues, blood vessels, and epidermal hairs were carefully removed before use. Porcine skin was mounted on the receptor compartment of a Franz cell with the SC side facing upwards into the donor compartment.

Freshly prepared 10% DMSO and 20% ethanol in 0.9% sodium chloride solution was used as the receptor solution for BT, and 0.9% sodium chloride solution was used as the receptor solution for TM. All were maintained at 37 °C under constant stirring. Once the skin temperature had equilibrated to 36 ± 1 °C, 1 mL of the treatment solution or vehicle was applied to the donor compartment. A volume of 1ml of receptor solution was collected at 30 min, 1, 2, 4, 6, and 8 h, and replaced with an equal volume of fresh temperature-equilibrated medium.

At the corresponding sampling time points, skin samples were obtained after receptor solution collection and disassembly of Franz diffusion cells. The skin was trimmed and washed twice with 0.9% sodium chloride, then frozen and stored in a −80 °C freezer. All samples were analyzed using liquid chromatography–mass spectrometry (LC-MS). The number of replicate experiments was n = 6.

#### 2.7.2. Quantification of BT and Timolol in Plasma and Skin Tissue

Nine (three for each group) three-week-old rats were anesthetized and their dorsal hair was shaved. They were given topical 0.9% sodium chloride, 0.5% TM or 0.5% BT treatment on their dorsal skin. The anesthetic and drug application methods previously described in Section 2.6.1 were used. Following topical administration, three rats from each group were euthanized at each time point (1, 2, and 4 h). Blood was drawn via tail vein puncture or cardiac puncture and immediately transferred into an EDTA collection tube (Orsin, Shanghai, China). The blood sample was centrifuged at 4000 rpm for 10 min, and the plasma was separated and frozen on dry ice. Skin samples were carefully excised and rinsed twice in 0.9% sodium chloride solution and dried. Skin sample weights and plasma volume were documented before they were frozen and stored in a −80 °C freezer (Thermo Fisher Scientific, Waltham, MA, USA) until analysis.

#### 2.7.3. Ex Vivo Human Skin Absorption

With institutional ethical committee approval (SH9H-2019-T164-1) and under informed consent, normal human skin was obtained from rhytidectomy patients during surgery and minced with surgical scissors. Skin tissues were weighed and skin homogenate was prepared by mixing tissue samples with 0.9% sodium chloride three times its volume (3 µL per mg drug) using a bead mill homogenizer. Skin homogenates were placed in 5-mL centrifuge tubes with 1 µM BT or vehicle (0.9% sodium chloride) and incubated at 37 °C on a 60 r/min rotator for 1 to 4 h. After appropriate incubation, 50 µL solution sample was mixed with 150 µL acetonitrile and stored at −80 °C.

### 2.8. LC-MS Analysis

In a 1.5-mL centrifuge tube, a 100 µL sample was mixed with 300 µL acetonitrile and 200 ng/mL internal standard (tolbutamide) solution, vortexed for 3 min, and centrifuged at 14,000 rpm at 4 °C for 20 min. Supernatant (120 µL) was collected for mass spectrometry.

Sample drug concentration was detected by LC-MS-8030 (Shimadzu, Kyoto, Japan). The chromatographic column consisted of an ODS-C18 column (100 × 2.1 mm, 3.5 µm). The mobile phase consisted of solution A containing 0.1% formic acid and solution B containing 100% acetonitrile. The chromatographic conditions were: gradient elution, time: 0–1–2–3–7 min, A% = 90%–5%–5%–90%, flow rate: 0.2 mL/min; column temperature: 40 °C; injection volume: 10 μL; interface temperature 400 °C, DL temperature 250 °C, nebulizing gas flow 3 L/min, heat block temperature 400 °C, and drying gas flow rate 15 L/min.

### 2.9. Data Analysis

Compounds concentrations, standard curve calibration and quality control samples were determined using linear regression (Linear) based on the ratio of the peak area of the compound to the corresponding internal standard, with a weight factor of 1/×2. Data collection and processing of mass spectrometry instruments were carried out using Lab Solution software. Values are expressed as the mean ± SD. Where appropriate, data were statistically analyzed using either student t-tests or one-way ANOVAs. In all cases, the significance level was set at *p* < 0.05. Statistical analyses were performed using GraphPad Prism (Version 8.3.1, GraphPad Software, San Diego, CA, USA).

## 3. Results

### 3.1. Characterization and Toxicity Evaluation of Pro-Drug BT

The molecular weights of TM (432.492) and BT (386.51) were determined by an electrospray ionization mass spectrometry (ESI/MS) (Figure 1b,c). BT was characterized by 1H NMR spectroscopy. As shown in Figure 1d, the peaks at δ 1.6 ppm in 1H NMR spectrum were assigned to the methyl protons of butyryl group. The 1H NMR spectra also showed two signals of methylene of butyryl group at δ 2.3 and 2.8 ppm.

**Figure 2:**
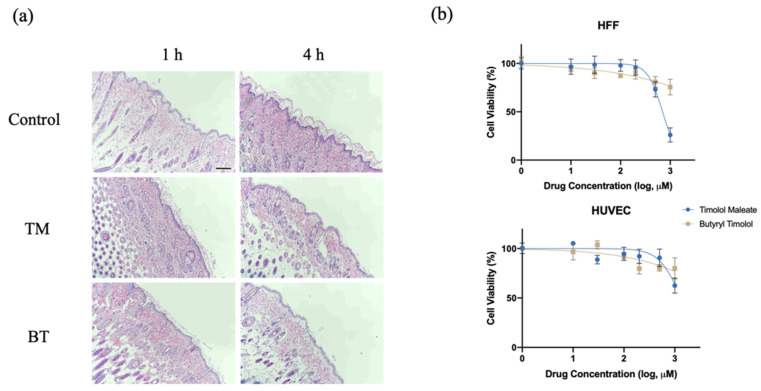
In vitro and in vivo toxicity evaluation of BT. (**a**) Histological examination of nude mouse dorsal skin stained with hematoxylin and eosin with no treatment (control), topical TM, and topical BT application for 1 and 4 h. Scale bar = 250 µm; (**b**) Cytotoxicity of TM and BT on normal human umbilical vein endothelial cells (HUVEC) and human foreskin fibroblasts (HFF) assessed by CCK8 assay. Values represent the mean ± SD (n = 3).

After topical application of 0.5% BT and 0.5% TM on a dorsal skin patch of a hairless rat for 1 and 4 h, the skin had no visible differences from that of the control group, and was without irritation or signs of dermal toxicity. Figure 2a shows histological examinations of rat skin after TM and BT application, revealing normal morphology and clear layers of the dermis, without signs of irritation or structural damage. An analysis of the cytotoxicity of BT on HUVEC and HFF-1 cells revealed that low concentrations of BT did not cause cellular death. Compared with the cytotoxicity of TM, BT exhibited an overall low cytotoxicity on normal human tissue cells (Figure 2b).

### 3.2. BT Exhibits Superior Skin Permeation and High Biotransformation

#### 3.2.1. BT Exhibits Superior Skin Permeation and Skin Retention Compared with That of TM Ex Vivo 

**Figure 3:**
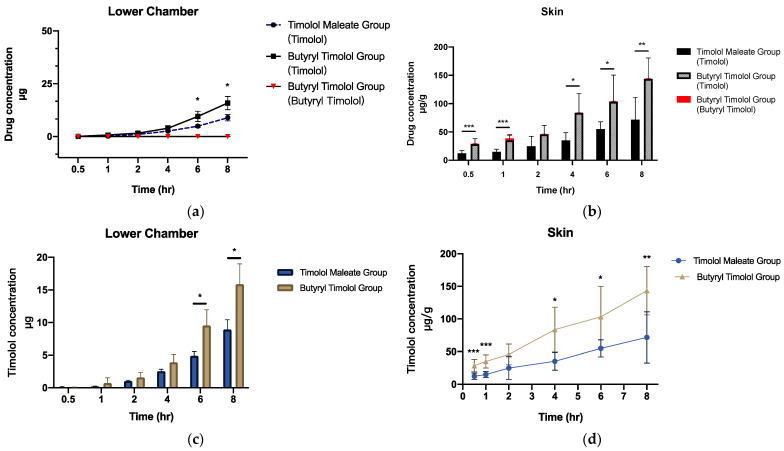
Ex vivo skin permeation and skin retention data of TM and BT application across porcine skin (**a**) cumulative flux (µg) and (**b**) skin retention (µg/g) of timolol and butyryl timolol after TM and BT application across time. (**c**) Cumulative flux (µg) and (**d**) skin retention (µg/g) of timolol alone after topical BT and TM application across time. All data are presented as the mean ± SD (n = 6). * *p* < 0.05; ** *p* < 0.01; *** *p* < 0.005.

When BT is applied onto the skin, this is expected to result in superior localized skin delivery to IH lesions. We investigated the drug release profile of BT by setting up a realistic ex vivo skin permeation study using porcine dorsal skin. The results predominantly demonstrated that timolol levels increased in the skin and dispersed over time. BT converted to timolol at a high rate, and a minute quantity of BT was detected in both cumulative flux and skin (Figure 3a,b). Cumulative ex vivo flux concentration of timolol for both TM and BT application (Figure 3c) revealed superior skin penetration ability of BT, compared to that of TM. Figure 3d shows BT application produces more timolol accumulation in porcine skin through time, suggesting a much higher release profile of timolol than BT.

#### 3.2.2. BT Demonstrates High Skin Retention but Low Systemic Absorbance In Vivo

**Figure 4:**
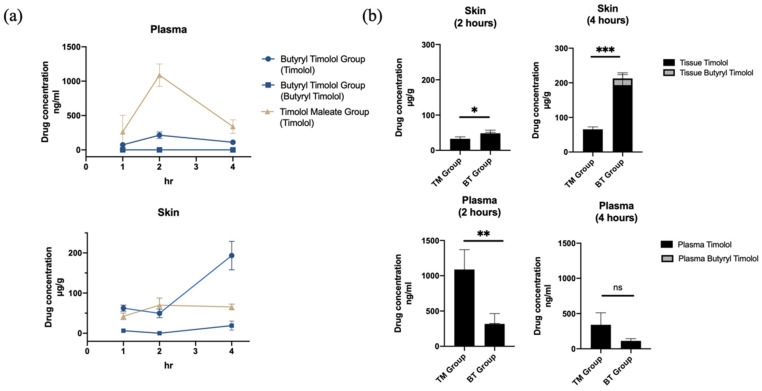
In vivo skin absorption data and plasma concentration of topical TM and BT application. (**a**) Drug concentrations in skin (µg/g) and plasma (ng/mL) over 4 h of TM and BT application. (**b**) Timolol and butyryl timolol levels detected in skin and plasma after 2 and 4 h of topical TM and BT. * *p* < 0.05; ** *p* < 0.01; *** *p* < 0.001; ns, not significant.

The systemic in vivo pharmacokinetics of drug absorption through the skin was investigated in three-week old rats. Figure 4a shows the comparison of mean timolol and BT skin tissue and plasma concentration time profiles from both application durations (1 and 4 h). In accordance with our ex vivo experiment results, there was a minimal amount of BT detected in both skin tissue and plasma following BT application, and timolol was predominately detected following the application of both drugs. Skin retention of the BT group continued to increase with time while plasma concentration remained low. By contrast, timolol skin absorption levels remained unchanged between 2 and 4 h post application in the TM group, and were significantly lower than that of the BT group (Figure 4).

Plasma concentrations of timolol reached their maximum at 2 h for both TM and BT groups and continuously decreased thereafter, but the BT group exhibited lower mean timolol plasma concentrations at its peak compared to that of the TM group (Figure 4b). BT displayed higher timolol skin retention, but had less systemic absorbance and thus, decreased the risk of drug side effects. Average (SD) skin and plasma drug concentrations for all groups are summarized in Table 1. 

#### 3.2.3. BT Is Converted into Timolol in a Time-Dependent Manner

**Figure 5:**
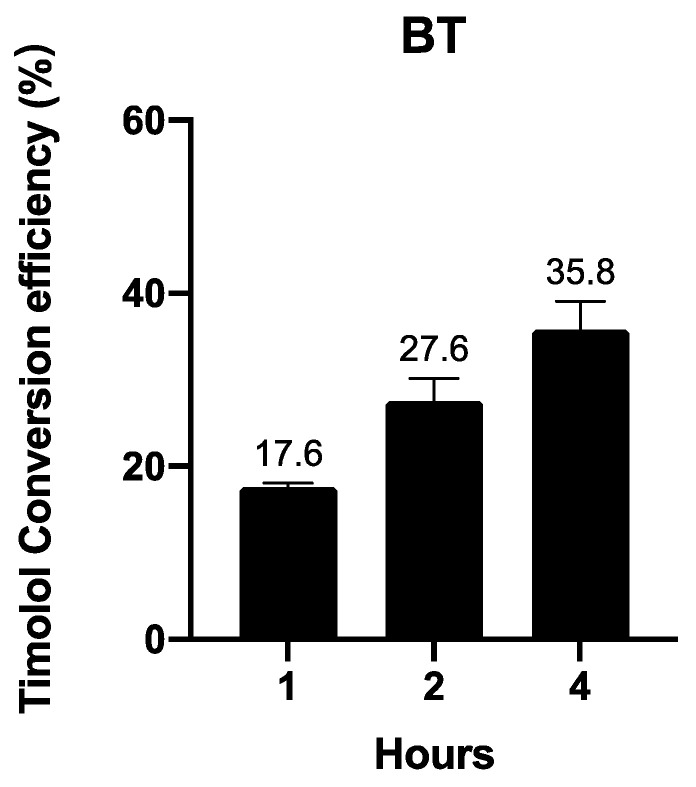
Ex vivo timolol conversion efficiency of 1 µM BT in human skin. Data are presented as the mean ± SD (n = 3).

In vivo and ex vivo experiments have uncovered large proportions of timolol instead of BT when BT is applied on animals. We further investigated BT’s conversion to timolol in human skin in an ex vivo experiment. Figure 5 shows that BT is converted into timolol upon contact with human skin in a time-dependent manner. 

### 3.3. BT Exhibits Similar IH Cellular Biocompatibility to TM 

**Figure 6:**
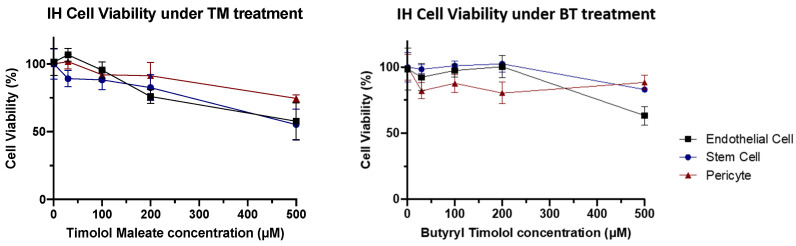
IH cytotoxicity assay. Cell viability tended to gradually reduce as higher concentrations of TM and BT.

When IH cellular toxicity was examined, cellular components of IH lesions (IH endothelial cells, IHEC; IH stem cells, IHSC; and IH pericytes, IHPC) showed no obvious decline in cellular viability at biological concentrations for both BT and TM. These results demonstrated that BT has a similar cytotoxic profile to that of TM in IH, both of which showed no obvious cell toxicity at low concentrations (Figure 6). 

## 4. Discussion

In this study, we found that BT, as a prodrug of TM, enhances timolol permeation and skin retention, facilitating timolol to pass through the skin and reach the deeper corners of the tumor while maintaining low systemic absorption. By employing ex vivo and in vivo animal and human tissue experiments, we have also demonstrated that BT is converted to timolol in the skin. BT’s biocompatibility in IH remains unchanged from that of TM. 

A prodrug is a chemical modification of the parent drug that is usually activated by a different environment from its parent drug (i.e., pH, oxidation–reduction, enzyme, etc.,). The physiological barrier to be penetrated by transdermal drug delivery is the skin surface (stratum corneum, SC), and the drug delivery route through the SC is via the intercellular lipid matrix. Therefore, better lipid solubility of transdermally administered drugs facilitates their passage through the SC [17]. Although SCs facilitate the penetration of lipophilic drugs, the epidermis and dermis are hydrophilic and thus act as a barrier to further penetration of lipophilic drugs into the lesion. Therefore, while lipophilic prodrugs can easily enter and diffuse through the SC, they cannot easily penetrate the epidermis and dermis. Esterases that hydrolyze esters are present in various tissues and organs, with specifically high activity in the epidermis and hair follicles [18]. Thus, once BT passes through the SC layer, its ester bond is hydrolyzed, transforming it into its parent drug, TM [19,20]. Hence, we hypothesized that BT would increase permeation of timolol through the skin, and thus will enable effective treatment of thick IH lesions.

To simulate skin of IH infants in our experiments, we used the skin of young animals (pigs and rats) without removal of the SC. We noticed that BT exhibited a high compatibility with skin tissue and IH cells, and showed cytotoxicity that was comparable to that of TM. This implies that the addition of butyryl chloride did not increase the toxicity of the drug. This toxicity evaluation result is important because TM is commonly used on infant skin, and BT will potentially be used to treat deeper lesions that TM could not. A recent trial demonstrated that higher plasma concentrations with IH drug use did not correlate with the hemangioma response, suggesting factors beyond skin penetration influence efficacy [21]. In our study, we demonstrated that skin permeation and retention of BT were superior to that of TM in both in vivo and ex vivo environments, whilst achieving a lower systemic absorption. This observation indicates that BT’s increased absorption and distribution are local. Importantly, by effectively reaching deeper parts of IH lesions, BT likely allows timolol to reach all corners of the lesion, thus effectively treating those IHs that had a high risk of developing into dermal sequelae. 

BT converts to timolol, which is the same active drug component as TM. However, a limitation of this study is that we have not yet found the exact mechanism by which timolol involutes IH tumors. IHSCs, IHPCs, and IHECs are the main components of IH in its proliferative phase. IHSCs possess a mesenchymal morphology, proliferate robustly, and can differentiate into IHECs and IHPCs to promote angiogenesis, and adipocyte development to generate involution [22,23,24]. There exist multiple mechanism-related speculations on why β-adrenergic receptor (AR) antagonists are effective at treating IH, although these theories have mainly been tested with propranolol. Lee et al. found that a significant decrease in blood perfusion was found within a short time period after propranolol administration, and thus speculated that the mechanism of propranolol’s effect on proliferating IH involves increased pericyte contractility, which in turn leads to local vasoconstriction and reduced local blood flow in IH [25]. Interestingly, Overman et al. reported that drug efficacy was independent of its β-AR blocker activity and was attributable to the direct targeting of the transcription factor SOX18, which, in turn, reduced hemangioma blood vessel formation [26]. Further, it is suggested that drugs display a pro-apoptotic effect on IHECs and pro-adipogenic effects on IHSC as the main contributing mechanisms of action [27,28,29]. However, a major concern regarding these experimental findings is that only higher concentrations (typically > 50 µM) of propranolol display significant effects. Such high concentrations are unlikely to be used in a clinical setting and therefore, are unlikely to exist in an IH tumor microenvironment. Another concern is that the majority of these results are obtained using propranolol hydrochloride, which is the first-line oral drug administered for life-threatening situations or used for treating severe IHs [30]. While TM, like propranolol hydrochloride is also a non-selective β-AR antagonist, its function cannot be inferred to be the same as observed by using propranolol hydrochloride. Clearly, more work is required to further understand the mechanism of action of TM for IH, in order to improve the drug efficacy. 

In our experiments, a minute amount of BT was detected throughout the course of experiments. To check if there was an error in the reading, we tested the reading of pure BT and recalibrated the standard curve. Most samples still had undetectable amounts of BT and a distinct quantity of timolol. Theoretically, the ester bond of BT is hydrolyzed when it comes in contact with the skin, where esterase hydrolyze esters are present, thus converting it into timolol. We speculate the rapid conversion from BT to timolol upon contact with skin tissue resulted in undetectable levels of BT in our samples. 

TM was first used as a topical application for IH in 2010 [31,32]. The mechanism by which timolol exerts its effect on IH is unknown, but it has since been widely accepted as an effective modality for treating superficial IH that produces minimal systemic side effects [33]. However, oral beta-blockers remain the recommended therapy for thick and deep IH lesions because of TM’s limited penetration. Therefore, a modification of the drug can provide a more targeted treatment, which is needed as an alternative to systemic therapy for localized, thick, and deep IH. BT can enhance the delivery of timolol to reach thicker and deeper lesions, accelerating the involution of these tumors that are at high risk of sequelae. Through this improved quality, BT could potentially help young children avoid dermal sequelae, disfigurement, and social isolation. 

## 5. Conclusions

Butyryl timolol (BT) is a synthetic prodrug that enhances timolol skin permeation. It exhibits low cytotoxicity and high biocompatibility with the skin, and a superior skin penetration and higher skin retention of timolol, compared to timolol maleate which is the traditional topical treatment for infantile hemangiomas. BT displays high conversion to timolol in the skin and perform its action as timolol. Its localized therapeutic effect can potentially treat thick or deep IH lesions and promote tumor involution, thus benefiting IH patients who are at risk of sequelae. 

## 6. Patents

A patent for the use of butyryl timolol for the treatment of infantile hemangioma is registered in the Chinese Patent Registry (CN201911268383.X).

## Figures and Tables

**Table 1:** In vivo skin and plasma drug concentrations after topical TM and BT application on SD rat skin. All data are presented as the mean (SD) (n = 3).

**Skin (ng/g)**		**BT Group**	**TM Group**
Timolol	4 h	193,469 (35,617)	65,638 (7046)
	2 h	41,205 (6678)	33,441 (5725)
Butyryl Timolol	4 h	19,148 (11,310)	10 (3)
	2 h	3698 (1386)	0 (0)
**Plasma (ng/mL)**		**BT Group**	**TM Group**
Timolol	4 h	111 (33)	339 (172)
	2 h	215 (85)	1087 (282)
Butyryl Timolol	4 h	0 (0)	0 (0)
	2 h	9 (5)	0 (0)

## Data Availability

The data presented in this study are available on request from the corresponding author.
